# First Evidence of Increased Plasma Serotonin Levels in Tako-Tsubo Cardiomyopathy

**DOI:** 10.1155/2013/847069

**Published:** 2013-09-23

**Authors:** C. Rouzaud Laborde, C. Delmas, J. Mialet-Perez, N. Pizzinat, C. Biendel-Picquet, N. Boudou, N. Dumonteil, O. Spreux-Varoquaux, D. Carrié, M. Galinier, A. Parini, O. Lairez

**Affiliations:** ^1^Inserm, Metabolic and Cardiovascular Disease Institute of Rangueil, 31432 Toulouse, France; ^2^Paul Sabatier University, 31062 Toulouse, France; ^3^Department of Pharmacy, University Hospital Center, 31059 Toulouse, France; ^4^Department of Cardiology, University Hospital of Rangueil, 1 avenue Jean Poulhès, 31059 Toulouse, France; ^5^Department of Pharmacology, Faculty of Medicine Paris-Ouest, 78000 Versailles St-Quentin-en-Yvelines, France; ^6^Department of Biology, Pharmacology Unit, General Hospital of Versailles, 78150 Le Chesnay, France; ^7^Cardiac Imaging Center, University Hospital Center, 31059 Toulouse, France; ^8^Department of Nuclear Medicine, University Hospital Center, 31059 Toulouse, France

## Abstract

*Background*. There is no data about the serotonergic activity during the acute phase of Tako-Tsubo Cardiomyopathy (TTC). The objective of our study was to investigate evidence of serotonin release from patients with TTC in comparison with patients with ST elevation myocardial infarction (STEMI) and healthy control subjects (HCS). *Methods and Results*. Plasma serotonin levels in 14 consecutive patients with TTC were compared with those in 14 patients with STEMI and 14 HCS. Plasma serotonin levels at admission were markedly higher in patients with TTC and STEMI as compared to HCS (3.9 ± 4.6, *P* = 0.02 versus control; 5.7 ± 5.6, *P* = 0.001 versus control; and 1 ± 0.4 ng/mL, resp.). There was no difference in serotonin levels between patients with TTC and those with STEMI (*P* = 0.33). *Conclusion*. This finding suggests that serotonin could participate to the pathophysiology of TTC.

## 1. Introduction

Tako-Tsubo cardiomyopathy (TTC) or stress-induced cardiomyopathy is a transient cardiac dysfunction that mimics myocardial infarction. Previous report suggests that exaggerated sympathetic stimulation could precipitate severe and reversible left ventricular dysfunction induced by emotional stress in patients without coronary disease [1]. Catecholamine-induced myocardial stunning has been postulated as a central mechanism of TTC, and transient increase of sympathetic nervous activity with increase of norepinephrine from the heart was suggested to participate to the TTC pathophysiology [2, 3]. Although a recent article reports a serotonin syndrome as an indirect cause of TTC [4], the role of serotonin in myocardial stunning after acute stressful event remains unknown.

The aim of our study was to investigate plasma serotonin release from patients with TTC in comparison with patients with ST elevation myocardial infarction (STEMI) and healthy control subjects.

## 2. Methods

### 2.1. Population

Between December 2011 and June 2012, 14 consecutive patients with TTC admitted to the Department of Cardiology, University Hospital of Rangueil, Toulouse, France, were prospectively included. As previously reported [5], TTC was diagnosed according to the following criteria: (1) acute cardiac event like chest pain, dyspnea, or syncope; (2) new ECG abnormalities (ST elevation, T-wave inversion, or abnormal Q-waves); (3) left ventricular systolic dysfunction with wall motion abnormalities extending beyond a single coronary perfusion bed (especially transient akinesia or hypokinesia of apical and midventricular segments with basal hyperkinesia); (4) absence of significant obstructive coronary artery disease (i.e., ≤50% luminal narrowing) or angiographic evidence of acute plaque rupture in the three major epicardial coronary arteries; (5) absence of myocarditis or typical ischemic late gadolinium enhancement on cardiac magnetic resonance imaging; and (6) absence of pheochromocytoma. These criteria are some of the criteria proposed by the Mayo Clinic for diagnosis of TTC [6].

During the same period, 14 patients with STEMI admitted for primary percutaneous coronary intervention and matched for age and sex were prospectively included as a control group. Diagnosis of STEMI was made according to the European Society of Cardiology Guidelines [7].

To compare plasma serotonin levels of patients with reference values, we used results from 14 healthy volunteers aged over 50 years included in the SERAOPI study (relation between SERotonin and AOrtic stenosis: a PIlot study) between April 2010 and November 2011 (study under publication). Protocol was unchanged and sampling was performed in the same conditions.

### 2.2. Clinical Testing

All patients were questioned about their medical history, demographic data, cardiovascular risk factors, and medications. All patients underwent a clinical examination, urinary sampling, and radial arterial and forearm venous blood sampling. Troponin C was measured on admission and at several times during the inhospital stay, and only maximum values were retained for analysis. Glomerular filtration rate was estimated by the Modification of Diet in Renal Disease (MDRD) formula.

As previously reported [5], a psychiatric assessment was performed using the Mini International Neuropsychiatric Interview (MINI) French version 5.0.0 based on the Diagnostic and Statistical Manual of Mental Disorders IV published by the American Psychiatric Association [8]. Interview was performed within the first 48 hours after admission. Among the 16 psychiatric disorders explored by the MINI, current or past major depressive disorder, generalized anxiety disorder, dysthymia, and posttraumatic stress disorder were selected for systematic psychiatric screening. Anxiodepressive disorders were defined by the presence of current or past major depressive disorder and/or generalized anxiety disorder. 

### 2.3. Assays of Plasma 5-HT

Radial arterial blood sample was realized at admission and removed on sodium citrate tubes, and a first centrifugation at 3000 g for 5 min at room temperature was performed to remove platelets. Superior 2/3 of supernatant was collected and centrifuged again for 5 min. Blood plasma was frozen at −80°C before HPLC analyses, as previously described [9]. Results were normalized by the platelets number.

### 2.4. Statistical Analysis

Continuous variables were tested for normal distribution using the Kolmogorov-Smirnov test and expressed as mean ± standard deviation. Comparison between two groups was performed using a *t*-test when the distribution of values was normal and a Mann-Whitney test when the distribution of values was not normal. Comparison between multiple groups was performed with analysis of variance (ANOVA) when the distribution of values was normal. Because the distribution of plasma serotonin level values was not normal, values were compared by pair using a Mann-Whitney test. Nominal values were expressed as numbers and percentages. An association between categorical variables was investigated by the Fishers exact test or *χ* ^2^ test as appropriate (effective ≤5 or >5, resp.). Differences were considered statistically significant for *P* values of <0.05. All analyses were performed using Statview (SAS Institute Inc., Version 5).

Oral informed consent was obtained from all patients, and the study was approved by our local institutional committee on human research.

## 3. Results and Discussion

Twelve out of 14 patients were female with a mean age of 70 ± 14 and 64 ± 11 years old (*P* = 0.23) for patients with STEMI and TTC, respectively. There was no difference for cardiovascular risk factors between the 2 groups. Patients with STEMI had lower creatinine clearance in comparison to patients with TTC (77 ± 22 versus 104 ± 32 mL/min; *P* = 0.02). For patients with STEMI, culprit lesion was on the right coronary artery, the anterior descending artery, and the circumflex artery for 6 (43%), 5 (36%), and 3 (21%) patients, respectively.

Physical or emotional stress was found in 10 (71%) and 1 (7%) patients with TTC and STEMI (*P* = 0.001), respectively. As previously reported [5], psychiatric interview screened more psychiatric disorders in patients with TTC with 11 (79%) and 3 (21%) patients with anxiodepressive disorders for TTC and STEMI groups (*P* = 0.007), respectively. None of the patients in both groups had antidepressive medication at admission.

Healthy control subjects were 9 (64%) males with a mean age of 62 ± 6 years old. There were 2 (14%), 3 (21%), and 3 (21%) subjects with current smoking habitus, arterial hypertension, and hyperlipidemia, respectively. None of the healthy control subjects had psychiatric disorder.

Characteristics of HCS and patients with STEMI and TTC are presented in Table 1.

**Figure 1:**
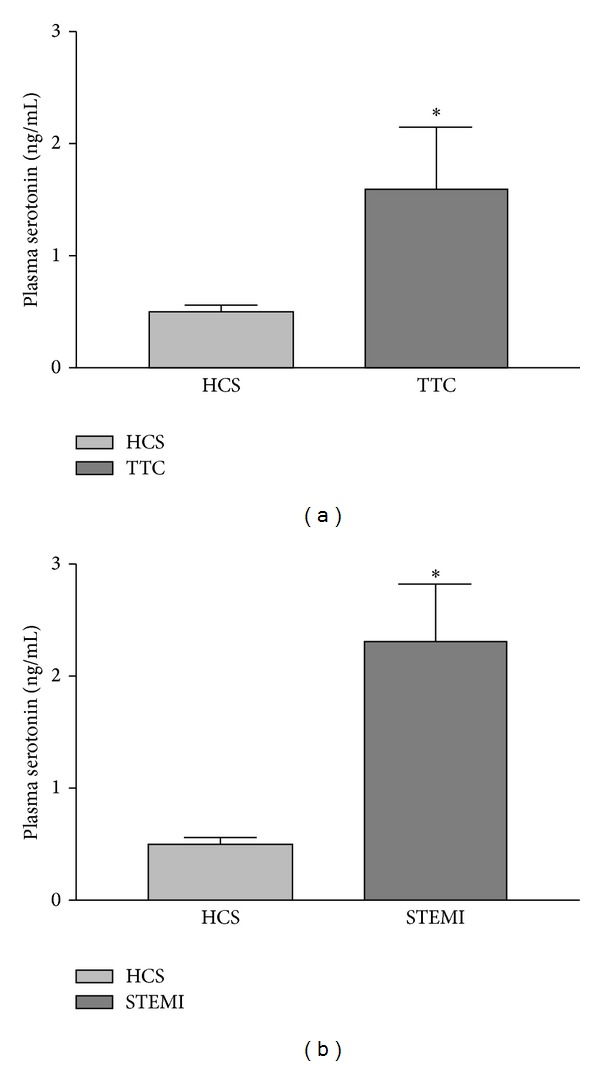
Plasma serotonin levels for patients and healthy control subjects. HCS: healthy control subjects; STEMI: ST elevation myocardial infarction; TTC: Tako-Tsubo cardiomyopathy. Plasma serotonin levels were normalized by platelets number. (a) Tako-Tsubo cardiomyopathy versus healthy control subjects; (b) ST elevation myocardial infarction versus healthy control subjects. *P < 0.05 versus healthy control subjects.

As shown in Figure 1, comparing results with healthy control subjects, both groups of patients (STEMI and TTC) showed an increase in plasma serotonin levels with values of 0.49 ± 0.07, 2.3 ± 0.51 (*P* = 0.008 versus control), and 1.59 ± 0.55 ng/mL (*P* = 0.03 versus control), respectively. There was no difference for plasma serotonin levels between patients with STEMI and patients with TTC (*P* = 0.30).

These results show for the first time that plasma serotonin levels are increased during the acute phase of TTC in the same range as patients with STEMI. These findings suggest that plasma serotonin could contribute to the pathophysiology of TTC, but the mechanism underlying the association between serotonergic stimulation and myocardial stunning remains unknown. In physiological situations, most of the circulating serotonin is stored in blood platelets after its biosynthesis in enterochromaffin cells. Large amounts of serotonin can be released in pathological situations associated with acute or chronic platelet activation. The increase in platelet aggregation, which could account for the raise in plasma serotonin, has been extensively reported in STEMI patients [10]. According to these findings, we found that plasma serotonin was significantly higher in STEMI patients as compared to control subjects. Our results are in agreement with a previous report showing a significant increase in plasma serotonin and its accumulation into the coronary thrombus in STEMI patients [11]. The mechanisms responsible for the increase of plasma serotonin in TTC patients remain to be defined. To date, there is no evidence for an increased platelet aggregation in TTC patients. However, our results, showing a strong increase in plasma serotonin, may be considered as the first indirect evidence supporting this hypothesis. 

Serotonin has a variety of effects on cardiomyocytes including inotropic effects [12], cardiomyocyte hypertrophy [13, 14], or apoptosis. Inotropic and hypertrophic effects are related to the classical serotonin mechanism of action involving stimulation of specific 5-HT receptors. On the other hand, the apoptotic effect, observed at higher 5-HT concentrations, is independent of receptor stimulation and needs hydrogen peroxide generation by the serotonin degrading enzyme monoamine oxidase A. Compared with healthy control subjects, TTC patients had supraphysiological levels of plasma serotonin in the same range as patients with STEMI. 

## 4. Limitations

The main limitation of our study is the absence of study of plasma serotonin kinetics and by-products levels. This lack of information does not permit as to determine if the increase of plasma serotonin levels is acute or chronic, nor the origin of plasma serotonin. As illustrated in Figure 1, our results show a wide range of plasma serotonin levels with a high variability of measures. This variability suggests that the increase of plasma serotonin levels is an acute and transient event with a fast return to physiologic levels. We can suppose that this acute increase of plasma serotonin levels is the consequence of a common step of platelet release. However, we cannot prove this hypothesis, which needs further studies.

## 5. Conclusion

Plasma serotonin levels are increased during the acute phase of TTC in the same range as patients with STEMI. This finding suggests that serotonin could participate to the pathophysiology of TTC. 

## Figures and Tables

**Table 1:** Characteristics of healthy control subjects and patients with ST elevation myocardial infarction and Tako-Tsubo cardiomyopathy.

	Healthy control subject	ST elevation myocardial infarction	Tako-Tsubo cardiomyopathy	*P* value
	*n* = 14	*n* = 14	*n* = 14	
Age (years)	62 ± 6	70 ± 14	64 ± 11	0.12
Female (*n* (%))	5 (35)	12 (86)	12 (86)	0.48
Body mass index (kg/m^2^)	26.1 ± 0.9	26.4 ± 5.1	24.3 ± 4.5	0.4
LVEF (%)	66 ± 2	47 ± 2	42 ± 3	<0.0001
Cardiovascular risk factors (*n* (%))				
Current smoking	2 (14)	3 (21)	2 (14)	0.99
Previous smoking	4 (28)	1 (7)	6 (43)	0.10
Hypertension	3 (21)	11 (79)	7 (50)	0.01
Diabetes	1 (7)	3 (21)	1 (7)	0.59
Hyperlipidemia	4 (28)	8 (57)	6 (43)	0.27
Obesity	0	6 (43)	5 (36)	0.02
Creatinine clearance (mL/min)	96 ± 6	77 ± 22	104 ± 32	0.02
Psychiatric diagnosis (*n* (%))				
Current or past major depressive disorder	4 (28)	3 (21)	10 (71)	0.02
Generalized anxiety disorder	0	0	6 (43)	0.001
Biological samples				
Troponin peak (ng/mL)	NA	124 ± 125	3 ± 3	0.002
Hemoglobin (g/dL)	14.3 ± 0.3	12.3 ± 1.3	12.0 ± 3.8	0.001
Platelets counts (1000/mL)	210 ± 10	251 ± 79	262 ± 106	<0.0001

Proportions are tested by Fisher's exact test or *χ* ^2^ test as appropriate (effective ≤5 or >5, resp.), and quantitative values are tested by analysis of variance (ANOVA test).

LVEF: Left ventricular ejection fraction.
